# Association of recent antibiotic exposure and coronary artery lesions in Kawasaki disease: nationwide study

**DOI:** 10.3389/fped.2024.1467288

**Published:** 2024-11-01

**Authors:** Hideto Ansai, Masaki Yamada, Hiroshi Masuda, Ken-Ichi Imadome, Mayumi Yashiro, Magali Noval Rivas, Moshe Arditi, Yosikazu Nakamura, Jun Abe

**Affiliations:** ^1^ Department of General Pediatrics and Interdisciplinary Medicine, National Center for Child Health and Development, Tokyo, Japan; ^2^ Department of Advanced Medicine for Viral Infections, National Center for Child Health and Development, Tokyo, Japan; ^3^ Department of Public Health, Jichi Medical University, Tochigi, Japan; ^4^ Department of Pediatrics, Division of Infectious Diseases and Immunology, Guerin Children’s at Cedars-Sinai Medical Center, Los Angeles, CA, United States; ^5^ Department of Biomedical Sciences, Infectious and Immunologic Diseases Research Center (IIDRC), Cedars-Sinai Medical Center, Los Angeles, CA, United States

**Keywords:** Kawasaki disease, antibiotics, gut microbiota, coronary artery lesions, nationwide surveillance

## Abstract

**Objectives:**

To investigate the relationship between recent antibiotic exposure and the development of coronary artery lesions (CALs) during the clinical course of Kawasaki disease (KD).

**Design:**

Data were obtained from the 25th nationwide epidemiological survey of KD conducted in Japan from 2017 to 2018. Baseline characteristics and clinical course were compared between Antibiotics (+) and Antibiotics (–) groups.

**Setting:**

Nationwide survey of KD in Japan.

**Participants:**

KD patients were enrolled by response to a questionnaire sent to physicians working in pediatrics at hospitals with >100 beds.

**Exposure:**

Antibiotic exposure within one week before the first hospital visit as KD patients.

**Main outcome measures:**

The relationship between recent antibiotic exposure and the development of coronary artery lesions (CALs).

**Results:**

Out of 28,265 KD patients, 12,918 (45.7%) received antibiotics. In KD patients who received antibiotics in the week before KD diagnosis, the frequency of coronary artery lesions (CALs) at each phase were significantly higher compared to those who did not receive antibiotics. In further analysis using propensity score matching, recent antibiotic exposure and the initial IVIG resistance were associated with CALs at the acute and the sequelae phase. After adjusting for the status of initial IVIG resistance, recent antibiotic exposure remained associated with CALs during the acute phase (adjusted OR 1.29, 95%CI 1.16, 1.43) and the sequelae phase (1.26, 95%CI 1.04, 1.52).

**Conclusions:**

These observations suggest that recent antibiotic exposure might be associated with higher frequency of CAL development in KD patients, possibly by altering the gut microbiota and diminishing beneficial bacteria.

## Introduction

Kawasaki disease (KD) is an acute childhood febrile illness identified by characteristic symptoms associated with systemic vasculitis (1). Although high-dose intravenous immunoglobulin (IVIG) therapy is highly effective for KD patients, 10% to 20% of KD patients do not respond to IVIG, and coronary artery lesions (CALs) develop in 5% of KD patients (2, 3). Hence, KD remains the most common cause of acquired heart disease among children in developed countries (3). The etiology of KD remains largely unknown, but given its clinicopathological features, such as fever and acute onset of symptoms, both infections and altered immune responses are considered to be potential contributors.

Changes in the intestinal microbiota composition have been implicated in the pathogenesis of several disorders, such as obesity (4), asthma (5), and cardiovascular diseases (6). Several studies using culture-based methods as well as metagenomics analysis indicate that compared with healthy controls, KD patients exhibit alterations in their fecal microbiota composition and dysbiosis, characterized by diminished relative abundance of beneficial bacteria, such as *Lactobacillus*, and increased abundance of *Streptococcus* species (7–12). Furthermore, because the acute febrile manifestation mimics infectious disease, patients with KD are often treated with antibiotics before clinical diagnosis of KD (13–16). Antibiotics alter the abundance, taxonomic richness, and diversity of the intestinal bacteria, and antibiotic-induced intestinal dysbiosis can persist after long-periods (17). However, little is known as to whether antibiotic exposure during the acute phase of KD impacts the pathophysiology and outcome of the disease. In an age- and sex-matched case-control retrospective study on 50 KD patients and 200 control subjects in Japan, Fukazawa et al. reported that there was an association between previous antibiotic exposure and the onset of KD, but no data were provided on severity or outcome of KD such as development of CAL (18). In that study, the median interval between the final dose of antibiotics and the onset of KD was 2.5 months, a period that is insufficient for restoration of the gut microbiota and complete resolution of dysbiosis, and the authors suggested that prior antibiotic use may contribute to the development of KD by affecting the intestinal microbiota in infants and young children (17).

A previous study from Canada showed that in hospitals where the KD caseload was low, the frequency of patients with incomplete manifestations was higher, and they were more likely to be treated with antibiotics than in high-caseload hospitals (19). However, other studies have reported that incomplete KD was not associated with a difference in antibiotic exposure (20, 21). In a study reported from Greece, patients with incomplete KD and previous antibiotic treatment were less likely to develop CALs (22). In contrast, a recent study by Downie et al. reported a significantly higher rate of prior antibiotic use during acute KD among complete IVIG non-responders compared to IVIG responders (23). Similarly, two additional studies reported that antibiotic use during acute KD affected responsiveness to IVIG and was associated with increased IVIG resistance (24, 25). Teramoto et al. showed that dysbiosis in KD patients, marked by an increase in pro-inflammatory *Ruminococcus gnavus* and a decrease in inflammation-suppressing *Blautia* in gut microbiome, might be a susceptibility factor by affecting microbial diversity and inflammatory responses (26). Taken together, these studies suggest that dysbiosis of the gut microbiota may be linked to the pathogenesis of KD. However, despite these reports, it remains unknown whether antibiotic exposure before KD diagnosis may be associated with a higher risk of developing CALs. To address this question we investigated the relationship between recent antibiotic exposure, and the development of coronary artery lesions (CALs) during the clinical course of KD, using a nationwide epidemiological survey of KD conducted in Japan from 2017 to 2018.

## Materials and methods

### Longitudinal Kawasaki disease database surveillance in Japan

Nationwide epidemiological surveys of KD have been conducted in Japan every two years since 1970. The present study was conducted using the database from the 25th national survey (2017–2018), in which questions regarding antibiotic exposure were adopted for the first time. We sent the questionnaire to physicians working in pediatrics at hospitals with >100 beds and at specialized pediatric hospitals.

### Cohort population and variable definitions

The baseline clinical data were extracted from the database as follows: sex, date of birth, complete KD or not, recurrent status, parental and sibling history of KD, day of the first hospital visit (defining the onset of fever as day 1), the day of initial IVIG treatment, response to initial IVIG and, if applicable, the types of additional therapy following the first-line treatment, e.g., additional IVIG, steroids, infliximab, immunosuppressive agents, and plasmapheresis. Complete KD was defined as having five or six major signs/symptoms or four major clinical features with CALs (27). Patients were divided into two age groups: less than one year and over one year at the onset of the disease. The data also included whether patients received antibiotic treatment within one week before the first hospital visit, referred to below as “recent antibiotic exposure,” and the presence of CALs during the three-time points of evaluation by echocardiography: Phase 1 (at the time of diagnosis), Phase 2 (within 30 days from the onset), and Phase 3 [sequelae (> one month)].

We excluded patients based on the following criteria: (i) missing data of antibiotic exposure, (ii) did not receive initial IVIG, (iii) received IVIG regimens other than 2 g/kg single dose or missing the data of IVIG dosage, (iv) KD diagnosis being made while in the hospital for another medical reason, (v) no treatment within ten days, (vi) missing the date of the initial IVIG.

Because antibiotics were not randomly used in this study population, selection bias could be a factor, so propensity score matching was done to assign participants to each group having been exposed to antibiotics or not.

### Statistical analysis

We first performed propensity score matching to balance the baseline characteristics of the two groups. The propensity score was calculated using multivariate logistic regression with matching factors (age, sex, recurrent status, KD status as of complete or incomplete KD, and day of the first hospital visit and initial IVIG treatment). Differences in the features of KD patients between the two groups were compared. Continuous variables were expressed as the mean (SD), and categorical data were expressed by number (%). In this study, the criteria for CALs were based on the definition from the Japanese Ministry of Health, Labor and Welfare, and we excluded coronary stenosis, myocardial infarction, and valvular lesions. A coronary artery dilatation was defined if the maximum internal lumen diameter was >3 mm in children <5 years old or >4 mm in children ≥5 years old. A coronary artery aneurysm was defined as a lumen size of 4 to 8 mm, and a giant coronary artery aneurysm was defined as a lumen size ≥8 mm.

We performed bivariate comparisons using the Mann-Whitney *U*-test for medians and the chi-square test for categorical variables. Univariable and multivariable logistic regression models were conducted to find the independent association with CALs; unadjusted and adjusted odds ratio (OR) with 95% confidence interval (95%CI) were calculated for each factor of the features of KD patients. The significance threshold was set at 2-sided *P* values <0.05 for all analyses. The IBM SPSS Statistics for Windows package, version.27 (IBM, Armonk, NY, USA) was used for all analyses.

### Ethics approval

This study was approved by the Bioethics Committee for Epidemiological Research, Jichi Medical University (9 October 2018, no. 18-070).

## Results

**Figure 1:**
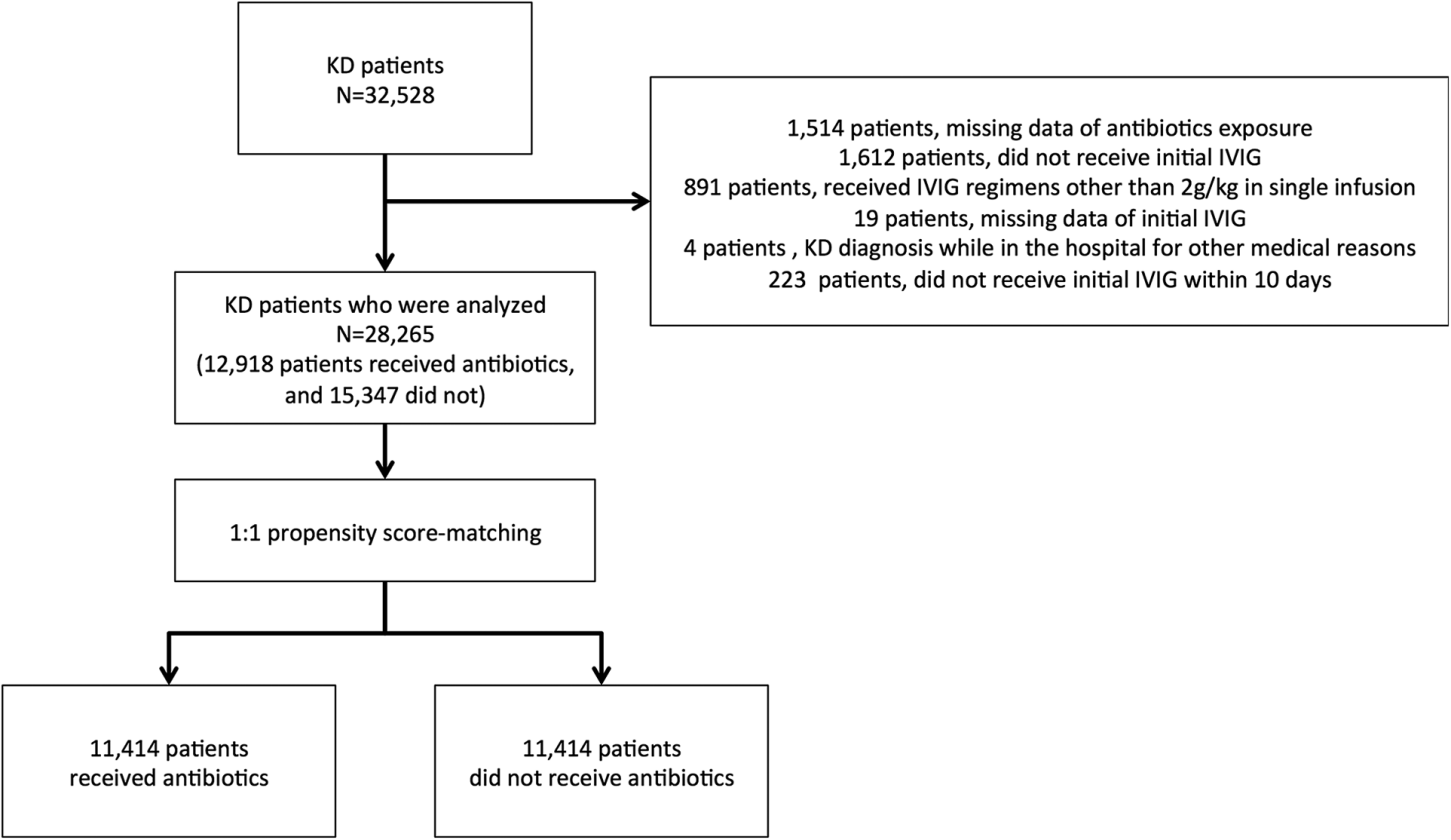
Schematic for study participants: KD, Kawasaki disease.

We asked 1,804 hospitals to participate in this study, and 1,357 (75.2%) responded to the survey. A total of 32,528 KD patients were included in the survey data. Among all patients, 1,514 were missing information regarding antibiotic exposure, 1,612 did not receive initial IVIG treatment, 891 received IVIG regimens other than 2 g/kg in a single infusion, and 19 were missing date of initial IVIG. There were four patients whose KD diagnoses were made while in the hospitals for other medical reasons and 223 who did not receive IVIG within ten days. Therefore, 4,263 patients were excluded from further analysis. Finally, 28,265 patients were chosen from this cohort survey, of whom 12,918 (45.7%) received antibiotics, whereas the rest did not. After the propensity score matching, we analyzed 11,414 patients in each group (Figure 1).

### Demographic and clinical data between the groups that received or did not receive antibiotics: antibiotics (+) vs. antibiotics (–)

The demographic data of patients in the two groups are shown in Table 1. Comparing the two groups, the proportion of children younger than one year was lower (12.9% vs. 23.3%), and the number of days of illness until the first hospital visit and the initial IVIG treatment was significantly longer in the group that received antibiotics than in the group that did not. Thus, we included the following factors, age, sex, recurrent status, KD status, and days of the first hospital visit and initial IVIG treatment in the propensity score matching. After propensity matching, the differences of these factors between the two groups were balanced, and the rate of initial IVIG resistance and the frequency of CALs at each phase were statistically higher in the antibiotic group. In observative data, the proportion of steroid use with 1st IVIG treatment was 12.7% vs. 13.6% between the antibiotics group and the non-antibiotics group respectively (*p* = 0.002), however after propensity matching, the differences were balanced to 13.6% vs. 14.2% (*p* = 0.22) (Table 1). In addition, the proportion of infliximab use was not different between the two groups in the observative data (2.8% vs. 2.5%, *p* = 0.12).

**Table 1:** Demographic characteristics between antibiotics(+) and antibiotics(–) groups in the observed data and the propensity-matched data.

	Observative data (*N* = 28,265)					Propensity-matched data (*N* = 22,828)				
	Antibiotics + (*N* = 12,918)		Antibiotics– (*N* = 15,347)		*P* value	Antibiotics + (*N* = 11,414)		Antibiotics– (*N* = 11,414)		*P* value
Male, No. (%)	7,373	57.1	8,832	57.5	0.42	4,763	41.7	4,882	42.8	0.11
Age <1 years, No. (%)	1,662	12.9	3,582	23.3	<0.001	1,631	14.3	1,616	14.2	0.78
Recurrent status, No. (%)	608	4.7	667	4.3	0.15	492	4.3	492	4.3	>0.99
Sibling history of KD, No. (%)	301	2.3	306	2.0	0.05	270	2.3	223	2.0	0.03
Parental history of KD, No. (%)	169	1.3	190	1.2	0.60	142	1.2	134	1.2	0.63
Complete KD, No. (%)	10,743	83.2	12,838	83.7	0.27	9,522	83.4	9,623	84.3	0.07
Dates of 1st visit, days, mean (SD)	4.26	±1.54	3.77	±1.49	<0.001	4.08	±1.27	4.05	±1.46	0.41
Dates of 1st IVIG, mean (SD)	5.23	±1.35	4.78	±1.31	<0.001	5.03	±1.22	5.03	±1.27	0.91
Corticosteroids with IVIG, No. (%)	1,644	12.7	2,094	13.6	0.002	1,557	13.6	1,621	14.2	0.22
Initial IVIG resistance, No. (%)	2,600	20.1	2,935	19.1	0.03	2,380	20.9	2,011	17.6	<0.001
CALs at phase 1	595	4.6	553	3.6	<0.001	521	4.6	377	3.3	<0.001
CALs at phase 2	1,045	8.1	1,058	6.9	<0.001	930	8.1	711	6.2	<0.001
CALs at phase 3	282	2.2	275	1.8	0.02	256	2.2	191	1.7	0.002

### Proportion of different sizes of CALs between antibiotics (+) and antibiotics (–) groups at each phase

We compared the proportion of different sizes of CALs before and after propensity matching between the two groups (Table 2). Although the proportion of giant aneurysms was not different between the two groups, the proportion of dilatations was higher in the Antibiotics (+) group at the time of diagnosis (Phase 1) and during the acute phase (Phase 2) before and after propensity matching, and in sequelae (Phase 3) after propensity matching. The proportion of aneurysms was higher in the Antibiotics (+) group in phase 1 before propensity matching, and in Phase 3 both before and after propensity matching.

**Table 2:** Different sizes of coronary artery lesions (CALs) between antibiotics(+) and antibiotics(–) groups before and after propensity-matching.

	Observative data (*N* = 28,265)					Propensity-matched data (*N* = 22,828)				
	Antibiotics + (*N* = 12,918)		Antibiotics– (*N* = 15,347)		*P* value	Antibiotics + (*N* = 11,414)		Antibiotics– (*N* = 11,414)		*P* value
CALs at phase 1										
Dilatations	552	4.3	523	3.4	<0.001	486	4.3	358	3.1	<0.001
Aneurysms	48	0.4	36	0.2	0.04	40	0.4	24	0.2	0.05
Giant aneurysms	4	0.03	5	0.03	0.94	3	0.03	3	0.03	>0.99
CALs at phase 2										
Dilatations	909	7.0	919	6.0	<0.001	808	7.1	613	5.4	<0.001
Aneurysms	124	1.0	124	0.8	0.17	111	1.0	87	0.8	0.08
Giant aneurysms	12	0.1	15	0.1	0.90	11	0.1	11	0.1	>0.99
CALs at phase 3										
Dilatations	200	1.5	198	1.3	0.06	181	1.6	136	1.2	0.01
Aneurysms	93	0.7	73	0.5	<0.007	83	0.7	51	0.4	0.005
Giant aneurysms	10	0.08	18	0.1	0.29	9	0.08	15	0.1	0.22

### Association between prior antibiotic exposure and development of CALs

In order to investigate the relationships between antibiotic exposure and the proportion of CALs, we conducted multivariable logistic regression analysis with the outcome being CALs at the time of diagnosis (Phase 1), during the acute phase (Phase 2) and in sequelae (Phase 3). Table 3 summarizes the coronary artery outcomes based on antibiotic exposure among the two groups. Before propensity score matching, there was a significant relationship between antibiotic exposure and the frequency of CALs. After matching for age, sex, recurrent status, KD status, and day of the first hospital visit and initial IVIG treatment, the frequency of CALs at each phase was still significantly higher in the Antibiotics(+) group (*P* < 0.001 at Phase 1 and 2, and *P* = 0.002 at Phase 3). Furthermore, the association at each phase was still significant after adjusting for resistance to initial IVIG treatment (adjusted OR 1.29, 95%CI 1.16. 1.43 in Phase 2, and 1.26, 95%CI 1.04, 1.52 in Phase 3). Stratified analysis according to complete KD or incomplete KD presentations was done to explore the relationship between antibiotic exposure and the development of CALs in these subgroups and showed a statistically significantly higher development of CALs at each phase in the Antibiotics(+) group in the complete KD group, while in the incomplete KD group the difference was significant only in the acute stage (Phase 2) (Table 4).

**Table 3:** Univariate and multivariate analysis for the development of coronary artery lesions (CALs) at each phase among patients exposed to antibiotics.

	Odds ratio	95% CI	*P* value
CALs at phase1			
Crude	1.29	1.15–1.46	<0.001
Adjusted for propensity	1.40	1.23–1.61	<0.001^a^
CAL at phase2			
Crude	1.19	1.09–1.30	<0.001
Adjusted for propensity	1.34	1.21–1.48	<0.001^a^
Adjusted for propensity and resistance to initial IVIG	1.29	1.16–1.43	<0.001^b^
CALs at Phase 3			
Crude	1.23	1.04–1.45	0.02
Adjusted for propensity	1.35	1.12–1.63	0.002^a^
Adjusted for propensity and resistance to initial IVIG	1.26	1.04–1.52	0.02^b^

^a^ Adjusted for sex, age, recurrent status, KD status (complete or incomplete KD), and days of the first hospital visit and initial IVIG treatment.

^b^ Adjusted for sex, age, recurrent status, KD status (complete or incomplete KD), days of the first hospital visit and initial IVIG, and resistance to initial treatment.

**Table 4:** Coronary artery lesions (CALs) between antibiotics(+) and antibiotics(–) groups, divided into kawasaki disease (KD) status (complete KD or incomplete KD).

Observative data	Complete KD (*N* = 23,581)					Incomplete KD (*N* = 4,684)				
	Antibiotics + (*N* = 10,743)		Antibiotics– (*N* = 12,838)		*P* value	Antibiotics + (*N* = 2,175)		Antibiotics– (*N* = 2,509)		*P* value
CALs at phase 1	551	5.1	517	4.0	<0.001	44	2.0	36	1.4	0.12
CALs at phase 2	967	9.0	999	7.8	<0.001	78	3.6	59	2.4	0.01
CALs at phase 3	267	2.5	261	2.0	0.02	15	0.69	14	0.56	0.57
Propensity-matched data	Complete KD (*N* = 19,145)					Incomplete KD (*N* = 3,683)				
	Antibiotics + (*N* = 9,522)		Antibiotics– (*N* = 9,623)		*P* value	Antibiotics + (*N* = 1,892)		Antibiotics– (*N* = 1,791)		*P* value
CALs at phase 1	484	5.1	350	3.6	<0.001	37	2.0	27	1.5	0.30
CALs at phase 2	861	9.0	670	7.0	<0.001	69	3.6	41	2.3	0.02
CALs at phase 3	244	2.6	180	1.9	0.001	12	0.63	11	0.61	0.94

## Discussion

This is the first nationwide study in Japan investigating the association between recent antibiotic exposure and the clinical characteristics and outcomes of KD. We found that recent prior antibiotic exposure one week before the diagnosis of KD may be independently associated with developing CALs in KD patients, possibly by altering the gut microbiota composition and changing the relative proportions of beneficial vs. detrimental bacteria.

There were several differences in the baseline and clinical characteristics between the Antibiotics (+) and Antibiotics (–) groups. In the Antibiotics (+) group, the proportion of ages under one year was smaller, and this may reflect that antibiotics were less likely to be prescribed for younger children compared with older children in Japan (28). Regarding a delay in the first hospital visit for KD and the initial use of IVIG in the Antibiotics (+) group (Table 1), symptoms of KD often mimic infectious disease, and the patients who received antibiotics might have required a longer observation period leading to a delay in the referral for hospitalization and, consequently, to a delay in the initial start of IVIG. After propensity matching, the above imbalances in the baseline factors were corrected, however the frequency of initial IVIG resistance and, more markedly, the proportion of CALs remained higher in the Antibiotics (+) group than in the Antibiotics (–) group. More importantly, the proportion of moderate-sized aneurysms was significantly elevated in the sequelae phase in Antibiotics (+) group compared to the Antibiotics (–) group after propensity matching, suggesting a strong association of antibiotic exposure and the development of CALs in KD. Early administration of initial IVIG was previously reported to be associated with initial IVIG resistance, and resistance to initial IVIG is a risk factor for developing CALs (3). In our cohort, the proportion of initial IVIG resistance was higher in the Antibiotics (+) group, although the timing of the first dose of IVIG was delayed.

Although no specific and reproducible stool microbiota signature has yet been observed in KD patients, increasing evidence suggests that dysbiosis and alterations in the intestinal microbiota could potentially influence the severity and outcome of KD. Indeed, the participation of the gut microbiota in the development and progression of KD cardiovascular lesions has been demonstrated in an experimental mouse model of KD vasculitis (29). Kaneko et al. proposed that dysbiosis characterized by reduced butyrate-producing bacteria in the gut microbiome could contribute to the development of KD by disrupting the balance of Th17/Treg cells, and potentially triggering hypercytokinemia (30). In addition to changes in the composition of the gut microbiota in KD patients, they also reported reduced levels of fecal short-chain fatty acids (SCFAs), such as butyrate, during acute KD (30). Bacteria-produced SCFAs such as buytrate are considered beneficial, as they promote intestinal barrier function and improve cardiovascular inflammation (31). Recently, a retrospective case-control study with 17,818 KD patients and 89,090 matched-control subjects revealed that antibiotic usage within the past 6 or 12 months may be associated with the development of KD among children, and that this association was most prominent in children who received 3 or more types of antibiotics within 12 months from developing acute KD (32).

Antibiotic exposure has the powerful effect of skewing the composition of the intestinal microbiota, and we hypothesize that prior antibiotic exposure might be associated with increased severity of KD and the development of CALs due to aberrant changes in the intestinal microbiota of KD patients (8). In addition, microbiota-host interactions could modulate the production of inflammatory cytokines (33). Interestingly, using a metagenomic approach, Kinumaki et al. revealed that particular intestinal bacterial genera, including several *Streptococcus spp*., were highly abundant during the acute phase of KD. They concluded that these bacteria might be involved in the pathogenesis of KD (7). Notably, an earlier study by Takeshita et al. showed a significant loss of beneficial *Lactobacillus* in the gut microbiota of KD patients both with and without recent exposure to antibiotics (8), and Chen et al. reported the presence of altered gut microbiota, particularly diminished SCFA-producing bacteria in a cohort of KD patients that were not previously exposed to antibiotics (12). Thus, further prospective metagenomics studies are required to identify the gut microbiota changes induced by antibiotic exposure that may drive the increase in vascular lesions in KD patients.

There are several limitations of the present study. First, because of the lack of space in the questionnaire, information regarding the details of the specific symptoms and indications that led to antibiotic use as well as the type of antibiotics used in these patients, as well as inflammation markers and BMI data were not collected in this national survey. This is an important caveat, as there might have been more severe symptoms of KD or other symptoms suggesting bacterial infections in the group that received antibiotics in the week prior to KD diagnosis. Indeed, patients who received antibiotics may have had symptoms similar to more severe illnesses, including sepsis. These patients in such severe clinical conditions usually require empiric antibiotic therapy and careful observation, which could result in a delay of IVIG treatment. However, after propensity matching, the days of illness until first dose of IVIG were not significantly different between the groups. Nevertheless, it is plausible that the patients in the Antibiotics (+) group could have had more severe illnesses and inflammation than those in the Antibiotics (–) group, and may represent a different subset of KD patients more predisposed to developing CALs. Therefore, questions regarding symptom severity, inflammatory markers and BMI information should be included in the questionnaire for future nationwide surveys in Japan. Second, there may be other confounding factors for severity of disease or risk factors that may influence the development of CALs besides those included in our survey and listed above. We used factors known to impact the development of CALs, so a selection bias of those predisposing factors may exist. A recent study reported that overweight/obesity may be an independent risk factor for CAL among KD patients (34). Third, we defined complete KD as having five or six major signs/symptoms or having four major clinical features together with CALs, so that patients who satisfied the definition of complete KD were more likely to be associated with the development of CALs. It is important to note that the diagnostic criteria for complete KD vary depending on the country. However, the number of patients that had complete KD by our definition was similar between groups. Finally, because this is a cross-sectional analysis of a nationwide survey study, our data can only indicate a potential association between prior use of antibiotics before the diagnosis of KD and the development of CALs. Nevertheless, the association reported here should stimulate future experimental animal and prospective studies investigating the use of antibiotics together with gut microbiome and metabolomic studies in KD patients to determine whether prior use of antibiotics is an independent predisposing factor for development of CAL.

## Conclusions

These observations suggest that recent antibiotic exposure might be associated with a higher frequency of CAL development in KD patients, possibly by altering the gut microbiota and diminishing beneficial bacteria. Prospective studies investigating the use of antibiotics and gut microbiota composition by metagenomic analysis are necessary to confirm whether prior use of antibiotics is an independent predisposing factor for CAL development in KD patients.

## Data Availability

The datasets presented in this study can be found in online repositories. The names of the repository/repositories and accession number(s) can be found below: https://www.jichi.ac.jp/dph/inprogress/Kawasaki/.

## References

[1] Burns JC Kushner HI Bastian JF Shike H Shimizu C Matsubara T Kawasaki disease: a brief history. Pediatrics. (2000) 106(2):e27. 10.1542/peds.106.2.e27 10920183

[2] Newburger JW Takahashi M Beiser AS Burns JC Bastian J Chung KJ A single intravenous infusion of gamma globulin as compared with four infusions in the treatment of acute Kawasaki syndrome. N Engl J Med. (1991) 324(23):1633–9. 10.1056/NEJM199106063242305 1709446

[3] McCrindle BW Rowley AH Newburger JW Burns JC Bolger AF Gewitz M Diagnosis, treatment, and long-term management of Kawasaki disease. Circulation. (2017) 135(17):e927–99. 10.1161/CIR.0000000000000484 28356445

[4] Turnbaugh PJ Hamady M Yatsunenko T Cantarel BL Duncan A Ley RE A core gut microbiome in obese and lean twins. Nature. (2009) 457(7228):480–4. 10.1038/nature07540 19043404 PMC2677729

[5] Noval Rivas M Crother TR Arditi M. The microbiome in asthma. Curr Opin Pediatr. (2016) 28(6):764–71. 10.1097/MOP.0000000000000419 27606957 PMC5241015

[6] Witkowski M Weeks TL Hazen SL. Gut microbiota and cardiovascular disease. Circ Res. (2020) 127(4):553–70. 10.1161/CIRCRESAHA.120.316242 32762536 PMC7416843

[7] Kinumaki A Sekizuka T Hamada H Kato K Yamashita A Kuroda M. Characterization of the gut microbiota of Kawasaki disease patients by metagenomic analysis. Front Microbiol. (2015) 6:824. 10.3389/fmicb.2015.00824 26322033 PMC4531854

[8] Takeshita S Kobayashi I Kawamura Y Tokutomi T Sekine I. Characteristic profile of intestinal microflora in Kawasaki disease. Acta Paediatr. (2002) 91(7):783–8. 10.1111/j.1651-2227.2002.tb03327.x 12200903

[9] Fabi M D'Amico F Turroni S Andreozzi L Filice E Brigidi P Gut microbiota dysbiosis in childhood vasculitis: a perspective comparative pilot study. J Pers Med. (2022) 12(6):973. 10.3390/jpm12060973 35743758 PMC9224684

[10] Khan I Li XA Law B U KI Pan BQ Lei C Correlation of gut microbial compositions to the development of Kawasaki disease vasculitis in children. Future Microbiol. (2020) 15(8):591–600. 10.2217/fmb-2019-0301 32490694

[11] Esposito S Polinori I Rigante D. The gut microbiota-host partnership as a potential driver of kawasaki syndrome. Front Pediatr. (2019) 7:124. 10.3389/fped.2019.00124 31024869 PMC6460951

[12] Chen J Yue Y Wang L Deng Z Yuan Y Zhao M Yuan Z Tan C Cao Y. Altered gut microbiota correlated with systemic inflammation in children with Kawasaki disease. Sci Rep. (2020) 10(1):14525. 10.1038/s41598-020-71371-6 32884012 PMC7471315

[13] Smith PK Goldwater PN. Kawasaki disease in adelaide: a review. J Paediatr Child Health. (1993) 29(2):126–31. 10.1111/j.1440-1754.1993.tb00464.x 8489792

[14] Binder E Griesmaier E Giner T Sailer-Höck M Brunner J. Kawasaki disease in children and adolescents: clinical data of Kawasaki patients in a western region (tyrol) of Austria from 2003 to 2012. Pediatric Rheumatology. (2014) 12(1):37. 10.1186/1546-0096-12-37 27643389 PMC5350606

[15] Han SB Lee S-Y. Antibiotic use in children with Kawasaki disease. World J Pediatr. (2018) 14(6):621–2. 10.1007/s12519-018-0157-3 29713927

[16] Dominguez SR Birkholz M Anderson MS Heizer H Jone PN Glode MP Diagnostic and treatment trends in children with Kawasaki disease in the United States, 2006–2015. Pediatr Infect Dis J. (2019) 38(10):1010–4. 10.1097/INF.0000000000002422 31365480

[17] Dethlefsen L Huse S Sogin ML Relman DA. The pervasive effects of an antibiotic on the human gut microbiota, as revealed by deep 16S rRNA sequencing. PLoS Biol. (2008) 6(11):e280. 10.1371/journal.pbio.0060280 19018661 PMC2586385

[18] Fukazawa M Fukazawa M Nanishi E Nishio H Ichihara K Ohga S. Previous antibiotic use and the development of Kawasaki disease: a matched pair case-control study. Pediatr Int. (2020) 62(9):1044–8. 10.1111/ped.14255 32306442

[19] Lin YT Manlhiot C Ching JC Han RK Nield LE Dillenburg R Repeated systematic surveillance of Kawasaki disease in Ontario from 1995 to 2006. Pediatr Int. (2010) 52(5):699–706. 10.1111/j.1442-200X.2010.03092.x 20113416

[20] Anderson MS Todd JK Glode MP. Delayed diagnosis of Kawasaki syndrome: an analysis of the problem. Pediatrics. (2005) 115(4):e428–33. 10.1542/peds.2004-1824 15805345

[21] Juan CC Hwang B Lee PC Lin YJ Chien JC Lee HY The clinical manifestations and risk factors of a delayed diagnosis of Kawasaki disease. J Chin Med Assoc. (2007) 70(9):374–9. 10.1016/S1726-4901(08)70023-6 17908651

[22] Giannouli G Tzoumaka-Bakoula C Kopsidas I Papadogeorgou P Chrousos GP Michos A. Epidemiology and risk factors for coronary artery abnormalities in children with complete and incomplete Kawasaki disease during a 10-year period. Pediatr Cardiol. (2013) 34(6):1476–81. 10.1007/s00246-013-0673-9 23463134

[23] Downie ML Manlhiot C Latino GA Collins TH Chahal N Yeung RS Variability in response to intravenous immunoglobulin in the treatment of Kawasaki disease. J Pediatr. (2016) 179:124–30.e1. 10.1016/j.jpeds.2016.08.060 27659027

[24] Lao S Zhou T Kuo HC Zhong G Zeng W. Risk factors for coronary artery lesions in Kawasaki disease independent of antibiotic use in Chinese children. Front Public Health. (2022) 10:81713. 10.3389/fpubh.2022.817613 35602151 PMC9118346

[25] Lee Z-M Chu C-L Chu C-H Chang L-S Kuo H-C. Multiple intravenous antibiotics usage is associated with intravenous immunoglobulin resistance in Kawasaki disease. Pediatr Neonatol. (2022) 63:117–24. 10.1016/j.pedneo.2021.06.020 34716128

[26] Teramoto Y Akagawa S Hori S Tsuji S Higasa K Kaneko K. Dysbiosis of the gut microbiota as a susceptibility factor for Kawasaki disease. Front Immunol. (2023) 14:1268453. 10.3389/fimmu.2023.1268453 38022552 PMC10644744

[27] Ayusawa M Sonobe T Uemura S Ogawa S Nakamura Y Kiyosawa N Revision of diagnostic guidelines for Kawasaki disease (the 5th revised edition). Pediatr Int. (2005) 47(2):232–4. 10.1111/j.1442-200x.2005.02033.x 15771703

[28] Yoshida S Takeuchi M Kawakami K. Prescription of antibiotics to pre-school children from 2005 to 2014 in Japan: a retrospective claims database study. J Public Health. (2018) 40(2):397–403. 10.1093/pubmed/fdx045 28453710

[29] Jena PK Wakita D Gomez AC Carvalho TT Atici AE Narayanan M. The intestinal microbiota contributes to the development of immune-mediated cardiovascular inflammation and vasculitis in mice. bioRxiv [Preprint]. bioRxiv:2024.05.28.596258. (2024). 10.1101/2024.05.28.596258 PMC11985309 40026151

[30] Kaneko K Akagawa S Akagawa Y Kimata T Tsuji S. Our evolving understanding of Kawasaki disease pathogenesis: role of the gut Microbiota. Front Immunol. (2020) 11:1616. 10.3389/fimmu.2020.01616 32793240 PMC7393004

[31] van der Hee B Wells JM. Microbial regulation of host physiology by short-chain fatty acids. Trends Microbiol. (2021) 29(8):700–12. 10.1016/j.tim.2021.02.001 33674141

[32] Kim T-H Shin JS Kim SY Kim J. Association of previous antibiotics and Kawasaki disease. The Pediatr Infect Dis J. (2024) 43:643–50. 10.1097/INF.0000000000004335 38534913

[33] Schirmer M Smeekens SP Vlamakis H Jaeger M Oosting M Franzosa EA Linking the human gut microbiome to inflammatory cytokine production capacity. Cell. (2016) 167(4):1125–36.e8. 10.1016/j.cell.2016.10.020 27814509 PMC5131922

[34] Shi H Weng F Li C Jin Z Hu J Chu M Overweight, abesity and coronary artery lesions among Kawasaki disease patients. Nutr Metab Cardiovasc Dis. (2021) 31:1604–12. 10.1016/j.numecd.2021.01.015 33812731

